# Comparison of postsurgical clinical sequences between completely embolized and incompletely embolized patients with wide nicked intracranial aneurysms treated with stent assisted coil embolization technique: A STROBE-compliant study

**DOI:** 10.1097/MD.0000000000010987

**Published:** 2018-06-18

**Authors:** Zi-Qiang Cai, Shi-Hong Chai, Xiang-Lei Wei, Ke-Zeng You, Jiang Li, Ding-Mei Zhang

**Affiliations:** Department of Imaging, Linyi Center Hospital, Linyi, P. R. China.

**Keywords:** computed tomography angiography, enterprise stent-assisted coil embolization, intracranial wide-necked aneurysm, magnetic resonance angiography, therapeutic effect

## Abstract

The technique of stent-assisted coil embolization has been widely used in the clinic, while its efficacy and safety have yet to be evaluated. This study investigates the values of computed tomography angiography (CTA), magnetic resonance angiography (MRA), and digital subtraction angiography (DSA) in evaluating the Enterprise stent-assisted coil embolization in the treatment of intracranial wide-necked aneurysm.

A total of 578 intracranial wide-necked aneurysm patients confirmed by MRA + CTA + DSA examinations were included and treated with Enterprise stent-assisted coil embolization in this study. All patients were assigned into complete embolization (CE) group and incomplete embolization (IE) group according to the results of postoperative MRA + CTA + DSA examinations and Raymond grades. Hunt-Hess grades, incidence of complication and Glasgow Outcome Scale (GOS) grades of patients were investigated to assess the therapeutic effect of Enterprise stent-assisted coil embolization in intracranial wide-necked treatment. Multivariate logistic regression analysis was performed to assess risk factors for the therapeutic effect of Enterprise stent-assisted coil embolization in intracranial wide-necked aneurysm.

CTA images offered a better and clearer view than MRA and DSA images in both the CE and IE groups. Both the sensitivity and specificity of CTA were apparently higher than those of MRA. Patients in the CE group enjoyed a higher good GOS rate but a lower incidence of complication than those in the IE group. In Enterprise stent-assisted coil embolization treatment, the Hunt-Hess grade, hypertension, and size of artery aneurysm were independent factors affecting the therapeutic effect of Enterprise stent-assisted coil embolization in intracranial wide-necked aneurysm.

Compared with MRA, CTA shows a higher value in evaluating the therapeutic effect of Enterprise stent-assisted coil embolization for the treatment of intracranial wide-necked aneurysm, and can thus serve as an important means of predicting the therapeutic effect of endovascular intervention in treating patients with intracranial wide-necked aneurysm.

## 1 Introduction

The unruptured intracranial aneurysm is comparatively popular in the general population, occurring in about 3.2% of the adult population worldwide (mean age of 50 years), and it is increasingly discovered incidentally due to the widespread application of computed tomography (CT) and magnetic resonance imaging (MRI) scanning techniques.^[1,2]^ In recent years, the use of stents in treating intracranial aneurysms is gaining more and more recognition, and the application of stent-assisted coiling also attracts much attention.^[3]^ It is widely acknowledged that wide-necked aneurysms are hard to treat due to the geometrical shape unfavorable for further operations or surgery.^[4]^ Generally speaking, current endovascular treatments of wide-necked aneurysms include the balloon remodeling technique, liquid embolic materials, and stent-assisted coil embolization, which are capable of reconstructing and protecting the parent artery.^[5,6]^ Stent-assisted coil embolization, maturely established in clinical routine, refers to a therapeutic method which involves the application of a scaffold to connect the aneurysm neck with its following coiling through the stent interstices.^[7]^ Equipped with a fixed closed-cell design, a newly introduced Enterprise stent (nitinol stent, Codman & Shurtleff, Raynham, MA) has brought new hope in wide-necked aneurysm treatment.^[8]^

Traditional coronary angiography is regarded as a basic technique to assess patients with suspected coronary atherosclerosis.^[9]^ However, the introduction of multislice computed tomography angiography (CTA), whose diagnostic sensitivity and specificity are outstandingly high, has made it possible to conduct noninvasive imaging of coronary anatomy.^[10]^ CTA is a noninvasive volumetric imaging method without any arterial puncture or catheter manipulation.^[11]^ Magnetic resonance angiography (MRA) is widely recognized as a substitute for intra-arterial angiography since it is based on the identification of blood flow in the cerebral vessels and can prevent radiation and iodinated contrast exposure from appearing in CTA.^[11,12]^ In light of the fact that MRA only images a limited part of the whole coronary arteries for each double-oblique acquisition, it is relatively time-consuming under most circumstances.^[13]^ Both CTA and MRA have been employed as effective tools in evaluating intracranial aneurysms.^[11]^ Though sharing similar functions, many authors have tried to make comparisons in terms of their effectiveness and efficiency in treating intracranial aneurysms.^[14]^ Under this context, the present study aims to investigate the value of CTA and MRA in assessing the function of Enterprise stent-assisted coil embolization in treating patients with intracranial wide-necked aneurysm.

## 2 Materials and methods

### 2.1 Ethics statement

This study was approved by the Ethics Committee of Linyi Center Hospital, and all patients provided informed consents.

### 2.2 Study subjects

A total of 593 intracranial wide-necked aneurysm-suspected patients diagnosed in Linyi Center Hospital between January, 2007 and September, 2017 randomly received MRA + CTA + digital subtraction angiography (DSA) examinations, among which 578 patients (282 males and 296 females) with an average age of 18 to 83 years (48.37 ± 11.35) were definitely diagnosed with intracranial wide-necked aneurysm. All study subjects were treated with Enterprise stent-assisted coil embolization and retested by CTA, MRA, and DSA after surgery. According to the results with a combination of Raymond grades, the patients were classified into 2 groups: complete embolization (CE) group composed of 465 patients in grade I and incomplete embolization (IE) group consisting of 113 patients in grades II and III. The inclusion criteria were as follows: all patients were diagnosed with intracranial wide-necked aneurysm after DSA test; all patients were observed with spontaneous subarachnoid hemorrhage (SAH) accompanied with or without intracranial hematoma; the diameter of aneurysmal neck was over 4 mm and the ratio of the tumor size to the aneurysmal neck was less than 1.5; all patients did not suffer from any other hepatic, pulmonary, renal, or intracranial disease. The exclusion criteria were as follows: patients who had artery aneurysm and vascular malformation due to some trauma; patients who were accidentally found with unruptured intracranial aneurysm; patients without complete clinical data.

### 2.3 CTA, MRA, and DSA examinations

The 128-slice spiral CT (SOMATOM Definition AS; Siemens Medical Solutions, Forchheim, Germany) scanner was used, with parameters set as follows: voltage, 120 kV; electricity, 120 to 175 mA; revolving speed, 0.4 seconds; pitch, 0.8; field of view, 20 to 24 cm; reconstruction matrix size, 512 × 512; slice thickness, 5 mm. Patients were first treated with intracranial plain scan and then with CTA following the CTA procedures. An amount of 60 to 90 mL nonionic contrast media was intravenously injected to the patients using a high pressure injector at a speed of 4.0 mL/s, and the injector was then washed using 40 mL of normal saline. Data obtained from the images were reprocessed and transmitted to the A workstation, and the scanning images were also further processed. Siemens Trio Tim 3T or Philips Medical Systems Achieva, 1.5T scanner and the 3-dimensional time-of-flit (3D TOF) imaging were used to obtain 3D images of the intracranial segments of the bilateral internal carotid and vertebrobasilar artery. Diagnostic analysis was mainly conducted with 3D MRA images, while original images and MRI scanning images were comprehensively used for diagnostic assessment when necessary. The SIEMENS Artis dTA DSA machine (Siemens, Erlangen, Germany) was employed and the modified Seldinger method was conducted for puncturing through femoral artery. The catheters were inserted into internal carotids on the 2 sides and vertebral arteries into which nonionic contrast media was then injected for DSA test. Images on the front, flank, and dual oblique sides were taken and DSA rotational angiography was finished. The mask images and reinforced images were subsequently transmitted to workstation for 3D reconstruction.

### 2.4 Image analysis

With no idea about scanning conditions, 2 experienced radiologists were invited to evaluate the 3 types of images using blind method and their consensus was necessitated. Regarding the analysis of MRA, CTA, and DSA images, original images, MIP images, 3D images, and rotation technique could be comprehensively used as aids when necessary. In case of the occasion where positive numbers or positions were inconsistent in 3 types of images, relevant images of patients can be checked again to find causes for missed diagnosis or misdiagnosis.

### 2.5 Treatment regimen

General anesthesia was performed for patients with intracranial wide-necked aneurysm, with conventional systemic heparinization during the surgery. Seldinger method was conducted for puncturing through femoral artery and a 6F catheter sheath was placed inside. Considering the location of cerebral aneurysms and the condition of parent artery, appropriate angle, passage, Enterprise stent, and coil were carefully prepared. After the general anesthesia, the 6F ENVOY catheter was inserted into femoral artery and high-pressure heparin normal saline solution was continuously poured into the catheter to avoid the appearance of thrombose and air inside the catheter. The Enterprise stent was put at the optimal position, and the micro guidewire was inserted into parent artery till 2 ends of the catheter were 5 to 10 mm longer over and fully covered the neoplasia neck. Subsequently, the transmission guidewire was replaced by the exchange length guidewire to take out the Prowler Select Plus microcatheter and insert the coil-compatible microcatheter, which ensured better function of the coil. Mesh technology and parallel technique were both adopted for Enterprise stent-assisted coil embolization. After the surgery, cerebral DSA was again implemented to determine the right release position of stent, embolization degree, and blood flow of parent artery. Patients in the IE group underwent surgical treatment within 1 to 2 weeks. After surgery, all patients were observed in the intensive care units for 24 hours. They were required to receive antiplatelet treatment by orally taking 75 mg of clopidogrel (H20113353, Zhejiang Apeloa Jiayuan Pharmaceutical Co, Ltd, Dongyang, China) and 100 mg of aspirin (H51021384, Sichuan Sunnyhope Pharmaceutical Co, Ltd, Chengdu, China). After 3 to 6 months, the amount of clopidogrel was gradually reduced to 0 and the aspirin was solely taken for the lifetime. All intracranial wide-necked aneurysm patients were treated with the Enterprise stent as well as coils, saccules and other supplementary tools manufactured by Microvention company (New York).

### 2.6 Therapeutic evaluation

The therapeutic effect of arterial embolization was assessed on the basis of DSA results and graded according to Raymond grading: if the percentage of embolization area in the artery aneurysm reached 100%, the patient was regarded as grade I (completely embolized); if the percentage of embolization area in the artery aneurysm was between 90% and 100%, the patient was regarded as grade II (mostly embolized); if the percentage of embolization area in the artery aneurysm was no more than 90%, the patient was regarded as grade III (partly embolized). The quality of prognosis was graded following the Glasgow Outcome Scale (GOS) system: 5 points represent good recovery; 4 points represent mild disability; 3 points represent severe disability; 2 points represent vegetative state; 1 point represents death.

### 2.7 Indicators for further observation

The embolization degree, incidence of complication, and the impacts of gender, age, and medical history on the prognosis of patients were observed after Enterprise stent-assisted coil embolization treatment. The effects of Hunt-Hess grades,^[15]^ hypertension, and pathogeny on the therapeutic effect of Enterprise stent were also identified (Table 1).

**Table 1:**
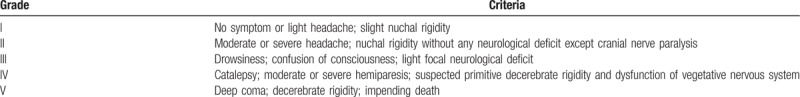
Hunt–Hess grade of intracranial aneurysm.

### 2.8 Follow-up and prognosis evaluation

The follow-up lasted 6 to 12 months, with a follow-up rate of 98.2%. It was conducted through outpatient service and telephone at the 3rd, 6th, and 12th months after Enterprise stent-assisted coil embolization.

### 2.9 Statistical analysis

All data were processed by SPSS 21.0 (IBM Corp, Armonk, NY). Enumeration data are presented as percentage and ratio. Comparisons between multiple groups were tested by *χ*^2^ test. Measurement data were expressed as mean ± standard deviation, and comparisons between 2 groups were analyzed by *t* test. With DSA results serving as the reference standard, the fourfold table was used to evaluate the significance of MRA and CTA in predicting the therapeutic effect of Enterprise stent-assisted coil embolization after surgery. Logistic regression analysis was carried out to assess factors influencing the therapeutic effect of Enterprise stent-assisted coil embolization on treating patients with intracranial wide-necked aneurysm. *P* < .05 was considered statistically significant.

## 3 Results

### 3.1 Clinicopathological features of intracranial wide-necked aneurysm patients between the CE and the IE groups

The differences in age, gender, history of coronary heart disease, location of artery aneurysm, smoking and drinking history, blood glucose level, and bleeding were of no statistical significance between the CE and IE groups (all *P* > .05). The ratio of subjects with aneurysm size no more than 15 mm in the CE group (81.51%) was significantly higher than that in the IE group (54.13%) (*P* < .05). The ratio of subjects with aneurysm size more than 15 mm in the CE group (18.49%) was significantly lower than that in the IE group (45.13%) (*P* < .05). The ratio of subjects with Hunt-Hess grades (0–III) in the CE group (80.86%) was significantly higher than that in the IE group (39.82%) (*P* < .05). The ratio of subjects with Hunt-Hess grades (IV–V) in the CE group (19.14%) was significantly lower than that in the IE group (60.18%) (*P* < .05) (Table 2).

**Table 2:**
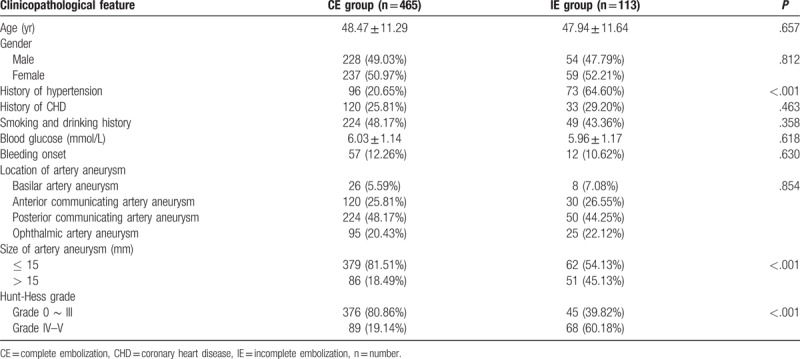
Clinicopathological features of intracranial wide-necked aneurysm patients between the CE and the IE groups.

### 3.2 CTA images provide a better and clearer view than MRA and DSA images

**Figure 1:**
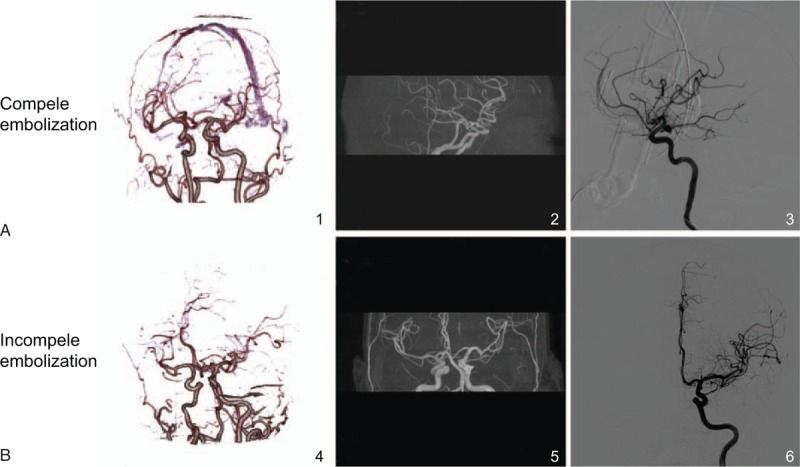
CTA, MRA, and DSA images of patients in the CE and IE groups 6 months after surgery. CTA images provide a better and clearer view than MRA and DSA images. A, CTA, MRA, and DSA images in the CE group: CTA image, no remain of artery aneurysm; MRA image, no visible remain of artery aneurysm; DSA image, no visible remain of artery aneurysm. B, CTA, MRA, and DSA images in the IE group: CTA image, little remain of artery aneurysm; MRA image, little remain of artery aneurysm; DSA image, little remain of artery aneurysm. CTA = computed tomography angiography, MRA = magnetic resonance angiography, DSA = digital subtraction angiography, CE = complete embolization, IE = incomplete embolization.

Next, we performed CTA, MRA, and DSA examinations and image analysis. In the CE group, CTA images provided a better and clearer view than MRA and DSA images, showing that there was no remain of artery aneurysm. In the IE group, CTA images also offered a better and clearer view than MRA and DSA images, showing that there was little remain of artery aneurysm (Fig. 1). Taken together, CTA images could provide a better and clearer view than MRA and DSA images.

### 3.3 Sensitivity and specificity of CTA are higher than those of MRA in the diagnosis of intracranial wide-necked aneurysm

According to the size of the aneurysm, the subjects were assigned into 2 groups: namely, patients with aneurysm no more than 15 mm and patients with aneurysm more than 15 mm. Among the patients with aneurysm no more than 15 mm, the sensitivity and specificity of CTA in the diagnosis of intracranial wide-necked aneurysm were 97.10% and 61.29% respectively, while those of MRA were 85.22% and 58.06% respectively (Table 3). Among the patients with aneurysm more than 15 mm, the sensitivity and specificity of CTA in the diagnosis of intracranial wide-necked aneurysm were 97.37% and 54.90% respectively, while those of MRA were 83.72% and 43.14% respectively (Table 4). The above results indicated that CTA could provide a higher sensitivity and specificity in detecting aneurysm.

**Table 3:**
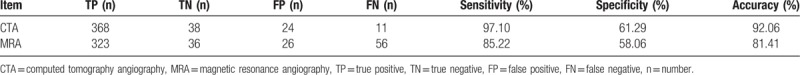
CTA provides higher sensitivity and specificity than MRA in detecting aneurysm no more than 15 mm.

**Table 4:**
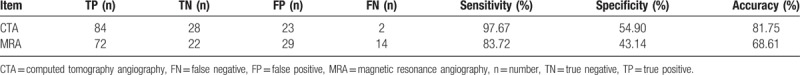
CTA provides higher sensitivity and specificity than MRA in detecting aneurysm more than 15 mm.

### 3.4 Patients in the CE group enjoy a higher good GOS rate but a lower incidence of complication than those in the IE group

Moreover, we compared good GOS rate in Hunt-Hess grade between the CE group and the IE group. Both the CE and IE groups showed a higher percentage of low Hunt-Hess grades than high grades (*P* *<* .05). Among patients in grade 0–III, the CE group had a significantly higher good GOS rate (97.34%) than the IE group (57.78%). Among patients in grade IV to V, the CE group also had a significantly higher good GOS rate (91.03%) than the IE group (52.94%) (Table 5). The incidence of complication in the CE group was obviously lower than that in the IE group (*P* *<* .05) (Table 6). It is concluded that patients in the CE group enjoyed a higher good GOS rate but a lower incidence of complication than those in the IE group.

**Table 5:**
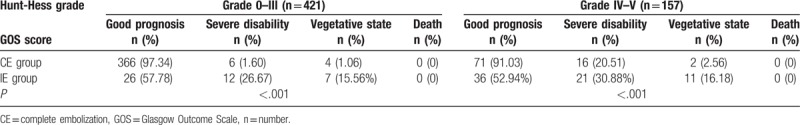
Patients in the CE group enjoy a higher good GOS rate than those in the IE group.

**Table 6:**
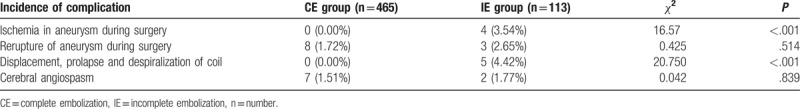
Patients in the CE group enjoy a lower incidence of complication than those in the IE group.

### 3.5 Hunt-Hess grades, history of hypertension, and aneurysm size are independent risk factors influencing the therapeutic effect of Enterprise stent-assisted coil embolization treatment in patients with intracranial wide necked aneurysms

At last, we performed multivariate logistic regression analysis to investigate risk factors for the therapeutic effect of Enterprise stent-assisted coil embolization in patients with intracranial wide necked aneurysms. The analysis results showed that the Hunt-Hess grades, history of hypertension, and aneurysm size were independent risk factors influencing the therapeutic effect of Enterprise stent-assisted coil embolization treatment in patients with intracranial wide necked aneurysms (all *P* *<* .05) (Table 7).

**Table 7:**
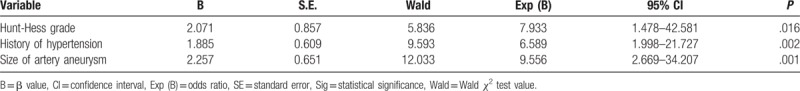
Hunt-Hess grades, history of hypertension and aneurysm size are independent risk factors influencing the therapeutic effect of Enterprise stent-assisted coil embolization treatment in patients with intracranial wide necked aneurysms.

## 4 Discussion

An estimated 2% to 3% of people across the world suffer from unruptured intracranial aneurysms, a disease primarily influenced by genetic factors as well as environmental factors.^[16,17]^ Fortunately, the recent 20 years have witnessed a substantial progress in the development of endovascular treatment for intracranial aneurysms, which is further epitomized by a change from an investigational therapy to a therapeutic approach for many lesions.^[18]^ The intracranial self-expanding stents like Enterprise stent have played a crucial role in promoting the coiling of wide-necked unruptured intracranial aneurysms.^[8]^ Although both MRA and CTA are powerful tools in the evaluation of intracranial aneurysms, only little evidence can offer convincing elucidation of their roles in assessing intracranial wide-necked aneurysm.^[11]^ Therefore, the present study conducted a series of experiments to identify the evaluation values of MRA and CTA in Enterprise stent-assisted coil embolization for intracranial wide-necked aneurysm. The results further suggested that CTA was of higher diagnostic value.

The comparisons of clinicopathological features of patients in the CE group and the IE group indicated that in terms of Hunt-Hess grade, there are more patients in grade 0 ∼ III but less patients in grade IV to V in the CE group than in the IE group. SAH, a life-threatening acute cerebrovascular disease, is most commonly and primarily caused by the rupture of intracranial aneurysm.^[19]^ Hunt-Hess grade refers to a grading system applied for the assessment of SAH severity, and a higher grade generally indicates a worse outcome.^[20]^ The prognosis of SAH shows close connection with the preoperative condition of patients (for example by Hunt-Hess grade).^[21]^ Therefore, patients in the CE group with fewer ruptured intracranial aneurysms had comparatively lower Hunt-Hess grade than those in the IE group. Besides, both the CE and IE groups had a larger proportion of patients with low Hunt-Hess grades (0–III) than high Hunt-Hess grades (III–IV). Additionally, the CE group enjoyed a higher good GOS rate among patients with both low and high Hunt-Hess grades and a lower incidence of complication than the IE group. A previous study once mentioned that patients with severe Hunt-Hess grade (IV–V) usually have fared poorly and approximately 20% to 30% of them were diagnosed with aneurysmal SAH.^[22]^ Some researchers also expounded on the fact that patients with low-grade aneurysms suffer from high mortality and disability rates.^[23]^ In cases where wide-neck aneurysms are involved, the protrusion degree of coils into the parent vessel was proven to increase the risk of complications such as thromboembolic formation.^[24]^ Thus, patients in the CE group were less likely to be attacked by complications.

When comparing the scanning results of patients with intracranial wide-necked aneurysm, CTA showed a better performance in judging whether there was any remain of intracranial aneurysm than MRA and DSA. DSA is a reference standard mainly followed to evaluate aneurysms after coiling, but it will bring patients with several risks, including contrast nephrotoxicity and ionizing radiation.^[17]^ MRA is restricted in use as the first line imaging especially in acute settings, because it is likely to become malfunctioned in night hours and in severe clinical presentations.^[25]^ Compared with MRA and DSA, CTA has better spatial resolution under the premises of contrast and radiation.^[26]^ It could also improve its temporal resolution by means of a time-resolved technique or a retarded acquisition.^[14]^ CTA enjoyed a higher degree of specificity and sensitivity than MRA in intracranial wide-necked aneurysm diagnosis. There is evidence elucidating that MRA is of lower sensitivity in assessing the feasibility of coiling compared with CTA.^[25]^ Moreover, it also suggested that MRA techniques presented lower sensitivity and specificity than DSA in the detection of any recanalization through the follow-up visit of coiled intracranial aneurysms, and the DSA is similar to CTA in terms of specificity and sensitivity,^[17,21]^ which supports our findings.

Finally, a multivariate logistic regression analysis indicated that Hunt-Hess grade, hypertension and aneurysm size were independent risk factors which influence the therapeutic effect of Enterprise stent-assisted coil embolization treatment. This conclusion can be further confirmed by many previous studies which acknowledged that Hunt-Hess grade, hypertension and aneurysm size all affect the efficacy of Enterprise stent-assisted coil embolization treatment.^[27–29]^

## 5 Conclusions

In conclusion, this study concluded that CTA has a higher value in evaluating the role of Enterprise stent-assisted coil embolization in treating patients with intracranial wide-necked aneurysm, implying that CTA can be considered as an important approach in assessing the endovascular intervention for intracranial wide-necked aneurysm treatment. However, further analyzing on the accuracy and efficacy of postoperative imaging would be needed in our subsequent study.

## Acknowledgment

The authors give their sincere appreciation to the reviewers for their helpful comments on this article.

## Author contributions

**Conceptualization:** Zi-Qiang Cai, Xiang-Lei Wei, Ding-Mei Zhang.

**Data curation:** Zi-Qiang Cai, Ke-Zeng You.

**Formal analysis:** Xiang-Lei Wei, Jiang Li.

**Investigation:** Shi-Hong Chai, Ding-Mei Zhang.

**Methodology:** Shi-Hong Chai, Ke-Zeng You, Jiang Li.

**Validation:** Xiang-Lei Wei.

**Writing – original draft:** Zi-Qiang Cai, Jiang Li, Ding-Mei Zhang.

**Writing – review & editing:** Zi-Qiang Cai, Shi-Hong Chai, Xiang-Lei Wei, Ke-Zeng You.
